# Microwave-Mediated Synthesis of Lead-Free Cesium Titanium Bromide Double Perovskite: A Sustainable Approach

**DOI:** 10.1021/acs.chemmater.3c03108

**Published:** 2024-02-02

**Authors:** Emmanuel Reyes-Francis, Carlos Echeverría-Arrondo, Diego Esparza, Tzarara López-Luke, Tatiana Soto-Montero, Monica Morales-Masis, Silver-Hamill Turren-Cruz, Iván Mora-Seró, Beatriz Julián-López

**Affiliations:** † Instituto de Investigación en Metalurgia y Materiales, Universidad Michoacana de San Nicolás de Hidalgo, Edificio U, Ciudad Universitaria, Morelia, Michoacán C.P. 58030, Mexico; ‡ Institute of Advanced Materials (INAM), Universitat Jaume I, Av. Sos Baynat, s/n, Castelló de la Plana 12071, Spain; § Unidad Académica de Ingeniería Eléctrica, Universidad Autónoma de Zacatecas, Jardín Juárez 147, Zacatecas Centro, C.P. 98000, Zacatecas 98000, Mexico; ∥ MESA+ Institute for Nanotechnology, University of Twente, Enschede 7500 AE, The Netherlands; ⊥ Department of Physical Chemistry, Polish Academy of Sciences, Warsaw 01-224, Poland

## Abstract

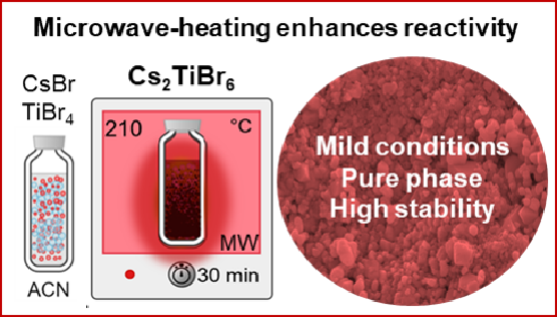

Theoretical studies have identified cesium titanium bromide (Cs_2_TiBr_6_), a vacancy-ordered double perovskite, as a promising lead-free and earth-abundant candidate to replace Pb-based perovskites in photovoltaics. Our research is focused on overcoming the limitations associated with the current Cs_2_TiBr_6_ syntheses, which often involve high-vacuum and high-temperature evaporation techniques, high-energy milling, or intricate multistep solution processes conducted under an inert atmosphere, constraints that hinder industrial scalability. This study presents a straightforward, low-energy, and scalable solution procedure using microwave radiation to induce the formation of highly crystalline Cs_2_TiBr_6_ in a polar solvent. This methodology, where the choice of the solvent plays a crucial role, not only reduces the energy costs associated with perovskite production but also imparts exceptional stability to the resulting solid, in comparison with previous reports. This is a critical prerequisite for any technological advancement. The low-defective material demonstrates unprecedented structural stability under various stimuli such as moisture, oxygen, elevated temperatures (over 130 °C), and continuous exposure to white light illumination. In summary, our study represents an important step forward in the efficient and cost-effective synthesis of Cs_2_TiBr_6_, offering a compelling solution for the development of eco-friendly, earth-abundant Pb-free perovskite materials.

## Introduction

Metal halide perovskite materials with ABX_3_ stoichiometry (for example, with A: CH_3_NH_3_^+^ or Cs^+^, B: Pb^2+^, X: I^–^ or Br^–^) and their derivates such as double metal halide perovskites with A_2_BX_6_ formulas have recently received significant attention, particularly for their demonstrated potential for photovoltaics and other optoelectronic applications. These materials have excellent properties like bandgap tunability, structural flexibility, and remarkable charge transport properties, resulting in excellent device performances.^1−4^ Power conversion efficiencies (PCEs) of lead-based perovskite solar cells (PSCs) reached up to 26.1% in 2023 equivalent to commercial solar cells and keep rising yearly.^5^ Furthermore, the affordability and accessibility of perovskite materials significantly encourage the development of the technology for commercial purposes.^6^ However, PSCs still face a long way to real-world applications due to essential concerns related to the toxicity of one of its fundamental components (lead) and their poor stability in the presence of heat, oxygen, moisture, electric field, and light.^7^ Thus, it is critical to develop stable lead-free perovskites from novel compositions for the advancement of the field.^8−10^

The abundant and environmentally friendly element titanium (Ti) is a significantly underexplored candidate with an enormous potential for the fabrication of sustainable perovskite technologies, especially the so-called Ti(IV)-based vacancy-ordered double perovskites.^11^ Among them, cesium titanium bromide (Cs_2_TiBr_6_), with a bandgap of 1.7–1.9 eV, demonstrates promising potential for solar applications,^12^ as evidenced by a combination of theoretical and experimental research.^13^ Its synthesis, however, remains challenging, with few reports so far. The reason lies on the high Lewis acidity of titanium(IV) reagents (usually TiBr_4_), which induces fast hydrolysis in the presence of oxygen (from residual oxygen, moisture, and ligands such as the typical oleic acid and oleylamine), leading to milky solutions (oxygenated titanium byproducts) a few seconds after reaction.^14^ Indeed, titanium halides (TiX_4_ with X: F, Cl, and Br) have been used to produce controlled-faceted and mesoporous TiO_2_ materials with enhanced catalytic activity. This was exemplified in pioneering works by Fornasiero et al.^15^ and Sanchez et al.^16^ The first work reporting the synthesis of Cs_2_TiBr_6_ powders involved a melt crystallization process at a high heating temperature (700 °C) for 72 h in an sealed quartz tube evacuated to ∼10^–6^ Torr.^13^ Later on, the same group prepared high-quality Cs_2_TiBr_6_ thin films using vapor-phase deposition of CsBr and TiBr_4_ at 200 °C.^14^ However, the process was extremely sensitive to reaction conditions (temperature, oxygen levels, etc.), and the reproducibility of single-phase Cs_2_TiBr_6_ films has been questioned.^17^ The high-energy mechanochemical synthesis by using ball milling has also been reported,^18^ but the product lacks significant photoluminescence and presents a high instability. The solution-based method is definitively a viable alternative to synthesizing this perovskite. However, the implementation of this specific methodology has been scarcely documented due to the difficulties involved with the high reactivity of dissolved titanium species and the toxicity of some reagents (HBr or HI for iodine relatives) and solvents (chlorobenzene, toluene, etc.).^19−21^

Additionally, some controversy has arisen over the stability of Cs_2_TiBr_6_ differently processed upon exposure to air. The solution synthesis developed by Euvrard et al. highlighted a strong instability of the powder, decomposing within a minute under ambient conditions at room temperature and moderate humidity levels.^22^ Another approach, involving a high-temperature vacuum melting process, reported similar instability.^23^ Conversely, nanoparticle-level synthesis techniques using the hot injection synthesis method have provided contrasting outcomes.^14,20^ Overall, this perovskite material demonstrated remarkable stability to light and temperature exposure but remains fragile to atmospheric conditions.^24^ Thus, the community should pursue novel strategies that increase the stability of this material with respect to the different degradation sources.

In this study, we present a novel, facile, and scalable microwave (MW)-mediated solution synthesis for Cs_2_TiBr_6_ perovskite. This method offers advantages in terms of simplicity, energy-cost, and speed in the synthesis of many other materials.^25−28^ Here, we develop a specific protocol for obtaining Cs_2_TiBr_6_, minimizing the handling of toxic reagents and avoiding the need for high-energy or high-vacuum setups. The use of acetonitrile as a solvent – with a low boiling point – enhances reaction kinetics and promotes the crystallization of the targeted Cs_2_TiBr_6_ phase. Furthermore, we examinate the material’s stability, assessing the impact of various stability factors, such as atmospherically conditions, temperature, oxygen, and light. The resulting material exhibited excellent stability to these parameters. This work provides Cs_2_TiBr_6_ double perovskites with improved stability by using homogeneous MW heating that accelerates reaction kinetics, guarantees high phase purity, and facilitates rapid product formation within a relatively short time.

## Experimental Section

### Chemicals

Cesium bromide (CsBr, 99.99%), titanium bromide (TiBr_4_, 99.99%), acetonitrile (ACN, 99.8%), and toluene (TOL, 99.8%) were purchased from Sigma-Aldrich. All chemicals purchased were used as received.

### Synthesis of Cs_2_TiBr_6_ Perovskite by MW-Mediated Process

**Figure 1:**
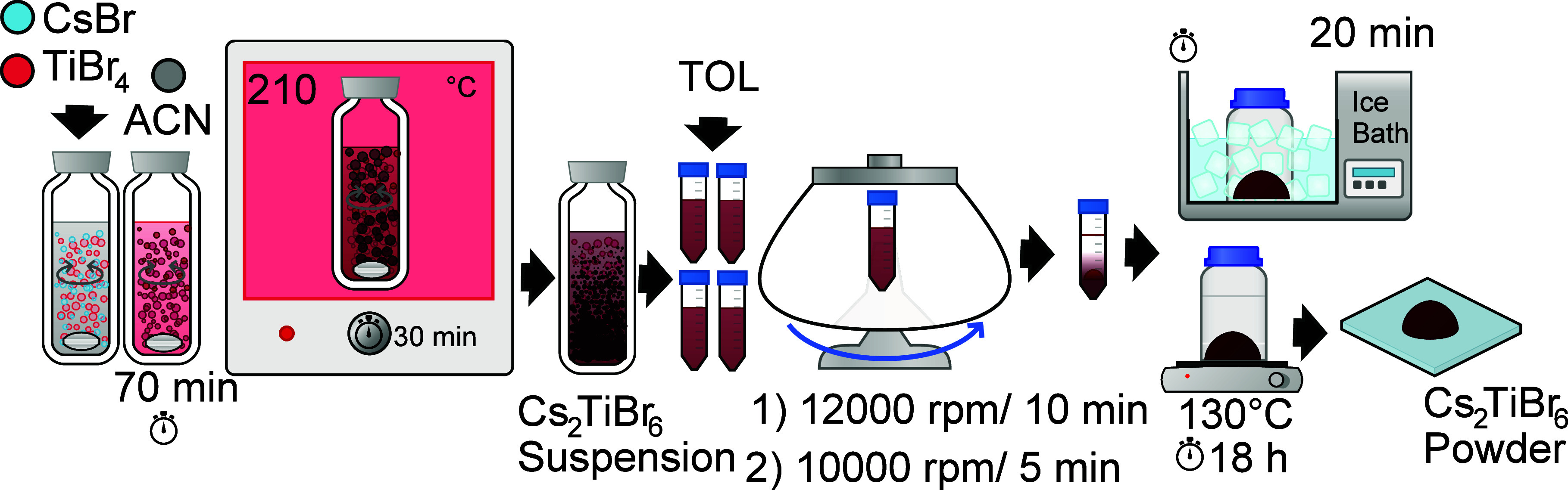
Schematic diagram of the MW-mediated synthesis of a Cs_2_TiBr_6_ powder.

Figure 1 describes the experimental approach to synthesizing Cs_2_TiBr_6_ powders by applying the MW methodology. In a general synthesis, 3.5 mmol of TiBr_4_ and 0.92 mmol of CsBr were weighted in the glovebox and loaded in a special glass vial for the microwave synthesis with 13 mL of ACN. After 70 min of stirring, the sealed glass was transferred to the microwave reactor (Anton Paar Monowave 400 monomode microwave reactor) and heated at 210 °C for 30 min. These conditions have been previously optimized from a screening study of reaction time and temperature. A deep red precipitate appeared after reaction. Then, 13 mL of toluene as antisolvent (1:1%v of ACN:TOL) was added to the resulting suspension. The precipitate was recovered by centrifugation at 12 000 rpm for 10 min. A washing procedure was carried out by adding 5 mL of TOL and centrifuging at 10 000 rpm for 5 min. The supernatant was discarded, and the precipitate was quenched in an ice bath for 20 min. Finally, the precipitate was transferred to the glovebox and heated at 130 °C for 18 h, obtaining the Cs_2_TiBr_6_ powder.

### Characterization Techniques

X-ray diffraction of the samples was performed on a D4 Endeavor diffractometer from Bruker-AXS, with a Bragg–Brentano (/2) geometry and Cu K radiation (0.15406 nm). The data was collected from 10° to 60° with a step scan of 0.05° and a counting duration of 0.5 s/step. The crystallite size was estimated by applying the Scherrer equation to the full width at half-maximum (fwhm) of the most intense peaks, with silicon as a standard for the instrumental line broadening. The morphology and composition of the samples were examined using a field-emission scanning electron microscopy (FE-SEM) microscope with an energy dispersive spectroscopy system INCA 250 (Oxford) and an acceleration voltage of 20 kV. The powders were directly deposited on a carbon film and coated with Au–Pt. The Fourier transform infrared spectra (FTIR) were obtained by using a Jasco FT/IR-6200 spectrometer equipped with an ATR Pron One device (Jasco). The ATR Pron One device used a diamond crystal, and the measurements were conducted with a resolution of 0.25 cm^–1^ over a spectral range of 4000 to 400 cm^–1^. Thermogravimetric analysis (TGA) measurements were performed on a NETZSCH TG 209F1 LIBRA instrument using a ramping rate of 5 °C/min from 30 to 600 °C, under nitrogen flow and oxygen flow. The absorption spectra were measured on a Cary 500 Scan UV–vis–NIR spectrophotometer (Varian) equipped with an integrating sphere and using BaSO_4_ as a blank reference. X-ray photoelectron spectroscopy (XPS) measurements were performed using an XM 1000 Al Kα monochromated X-ray source (1486.6 eV, fwhm = 0.26 eV) and an Omicron EA 125 energy analyzer with a pass energy of 50 eV, at a photoemission angle θ of 35°. An electron neutralizer beam is used to minimize binding energy shifts. During measurements, the pressure was <1 × 10^–10^ mbar. The samples were fixed with a copper double-sided conductive adhesive tape and analyzed as loaded. The spectra’ peak positions and width were fitted using a Gaussian–Lorentzian function (GL). A Shirley background was employed using the CasaXPS Software. Adventitious carbon was set at 485. Photoluminescence (PL) measurements were done in a home-built setup consisting of a 405 nm laser diode module, 100 mW (Matchbox series), and a StellarNetBLUE-Wave spectrometer coupled with a fiber optic.

## Results and Discussion

The microwave heating, as an alternative to convection heat treatment, provides faster heating when the system is able to efficiently absorb the applied electromagnetic radiation. Polar solvents (with high dissipation factor, tan δ) are usually the best media for MW-enhanced organic reactions and also nucleation/crystallization of inorganic or hybrid crystals (metal oxides, MOFs, etc.). Nevertheless, in this system, CsBr and TiBr_4_ reagents are highly polar species, and therefore, the charged ions can easily absorb microwave energy and convert it into thermal energy. Thus, it is possible to select a medium absorber solvent such as acetonitrile (tan δ: 0.062). Interestingly, acetonitrile exhibits fairly high dielectric constant (ε: 37.5), considering water as the solvent with the highest dielectric constant (ε: 80.4), but its dielectric loss is low (ε″: 2.375). As a result, the solvent molecules easily absorb radiation but hardly dissipate it, thus concentrating the energy in the polar reagents and enhancing reactivity. In addition, the boiling point of acetonitrile (82 °C) is bypassed in a matter of seconds, generating pressure in the reaction vessel. The experimental pressure at 210 °C reached 23.5 bar, which is higher than the pressure of pure acetonitrile estimated with Antoine’s equation (18.4 bar), meaning that some volatile species (i.e., generated HBr or even residual oxygen or water molecules from the solvent) are generated in the gas phase during reaction. All this explains the excellent reaction rate of the system in comparison to the previously reported solution processes to date.

**Figure 2:**
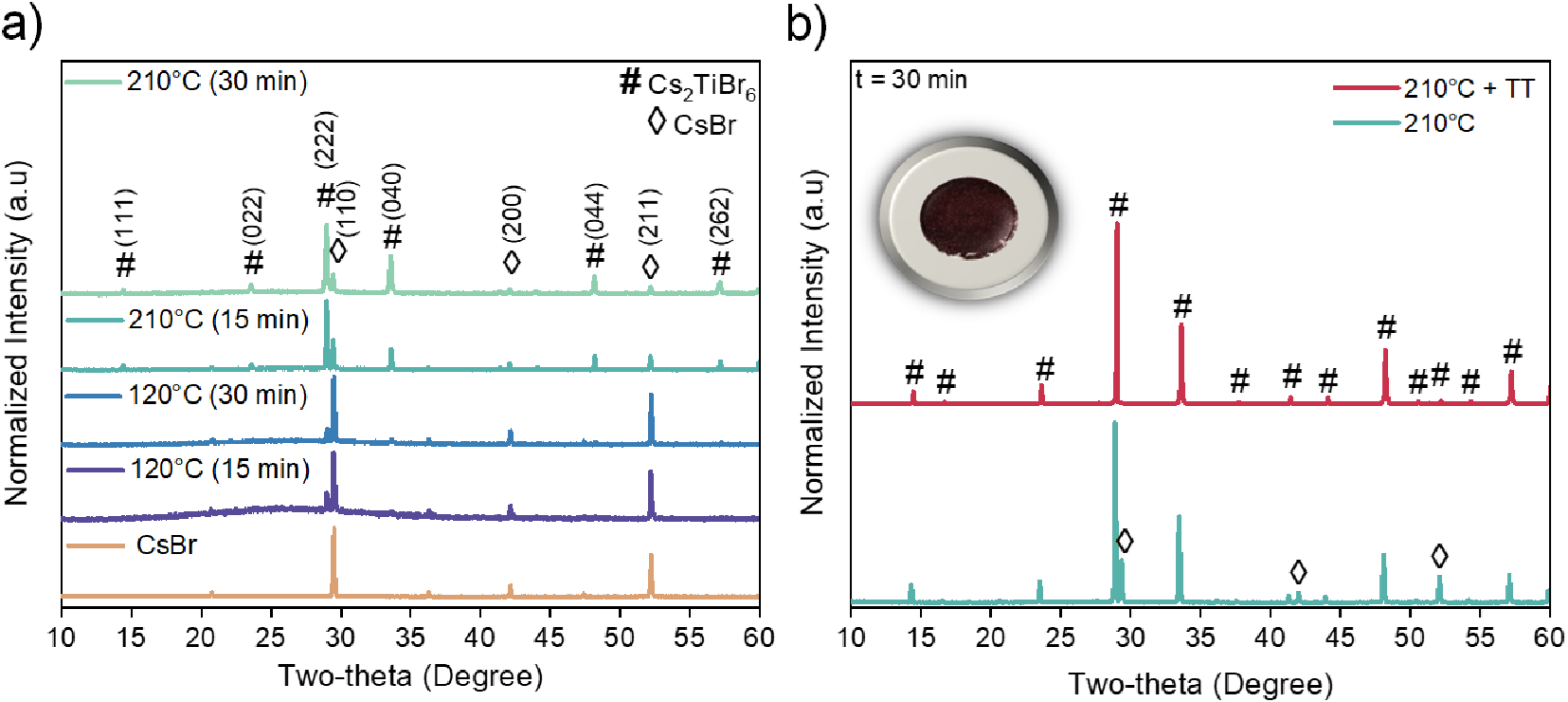
XRD patterns of the MW-synthesized Cs_2_TiBr_6_ powder: a) at different reaction time and temperature, and b) before and after the postsynthetic thermal treatment (TT) with an inset picture of the Cs_2_TiBr_6_ powder.

Figure 2a shows the X-ray diffraction patterns of the Cs_2_TiBr_6_ powders synthesized at different reaction times and temperatures. For simplicity, only the patterns of the extreme studied temperatures, i.e., 120 and 210 °C, for 15 and 30 min of MW reaction have been depicted.

We detected the presence of the targeted Cs_2_TiBr_6_ crystalline phase (*Fm3̅m* space group)^13,24^ and CsBr (ICDS card No 53 848) in all cases. At 120 °C, a high amount of CsBr appears in the solid, regardless of the reaction time. Nevertheless, the perovskite is the major phase at 210 °C, achieving an 80% yield of Cs_2_TiBr_6_ after 30 min of MW heating. Conversely, the intensity of CsBr experiences a progressive drop at a higher temperature. Figure S1a illustrates a comparative analysis of the relative percentage of both phases extracted from the intensity of the diffraction peaks according to the internal standard method (more details are included in the Supporting Information). Using Scherrer equation,^29,30^ the average crystallite sizes of both phases were estimated, ranging from 45 to 56 nm for the perovskite and from 53 to 60 nm for CsBr, between 120 and 210 °C reaction temperatures. Figure S1b plots this evolution under the selected conditions. We can see that the crystallite size of the emerging phase is relatively large (45 nm) even for the lowest temperature (120 °C) and time (15 min) and hardly evolves with increasing temperature and time. Thus, the crystalline domains remain in the nanometric range.

According to these results, the reaction time was standardized at 30 min. With this condition, we explored different synthesis temperatures between 120 and 210 °C. Their XRD patterns are shown in Figure S2. When analyzing the evolution of the Cs_2_TiBr_6_ and CsBr phases (Figure S3a) with increasing synthesis temperature, we observe a positive effect toward the formation of the double perovskite and reduction of CsBr. At 210 °C, after 30 min of reaction, around 80% of the material is converted to the targeted Cs_2_TiBr_6_, and its crystallite size (Figure S3b) reaches 56 nm. At this point, we must indicate that at 210 °C the microwave system was close to its pressure limit (30 bar), and we could not go further in temperature because of security reasons.

The presence of CsBr in the XRD patterns can be due not only to unreacted reagent but also to the degradation of the as-synthesized perovskite with time under atmospheric conditions (note that XRD measurements were performed several minutes, even hours, after the perovskite synthesis). This degradation is driven by the interaction of solvent and water molecules (from moisture) with the powder surface, leading to CsBr and amorphous titanium-based species. Then, a postsynthetic treatment was performed immediately after material’s recovery in order to avoid the degradation of Cs_2_TiBr_6_.^19^ This treatment was based on a mild annealing at 130 °C (slightly above the boiling point of water and toluene at 100 and 110.6 °C respectively) for 18 h in N_2_ atmosphere (inside glovebox) of the powders that were previously washed and quenched for 20 min in an ice bath. Under these conditions, the double perovskite is stabilized, and XRD patterns exhibited the peaks of Cs_2_TiBr_6_ as a single phase, see Figure 2b. As shown in the inset of Figure 2b, the color of the pristine Cs_2_TiBr_6_ perovskite material powder is dark red. The experimental XRD pattern fitted perfectly well to the pattern generated from modeling in silico according to the well established Cs_2_TiBr_6_ crystalline structure (Figure S4). Our computer simulations on the Cs_2_TiBr_6_ compound were conducted with density functional theory (DFT) and more specifically with the Vienna Ab Initio Simulation Package (VASP) program.^31,32^ DFT simulations were run at the PBEsol level of theory,^33^ where PBEsol stands for Perdew–Burke–Ernzerhof exchange-correlation functional revised for solids, using pseudopotentials.^34^ Bragg’s law was used to determine the lattice constant of the material from the XRD pattern. The obtained value was 10.63 ± 0.93 Å, which is consistent with the one reported in the literature.^24^

The morphological characterization of optimized Cs_2_TiBr_6_ powders was performed by SEM. A representative micrograph and EDS energy spectrum are shown in Figure S5. The images reveal that the powder is mainly formed by small agglomerates of Cs_2_TiBr_6_ nanocrystals with a submicrometer size in average. The EDS spectrum indicates that Cs, Ti, and Br constitute 22.1%, 11.3%, and 66.6% of the atomic weight ratio, respectively, aligning with the elemental ratio of the perovskite, which is 2:1:6.

**Figure 3:**
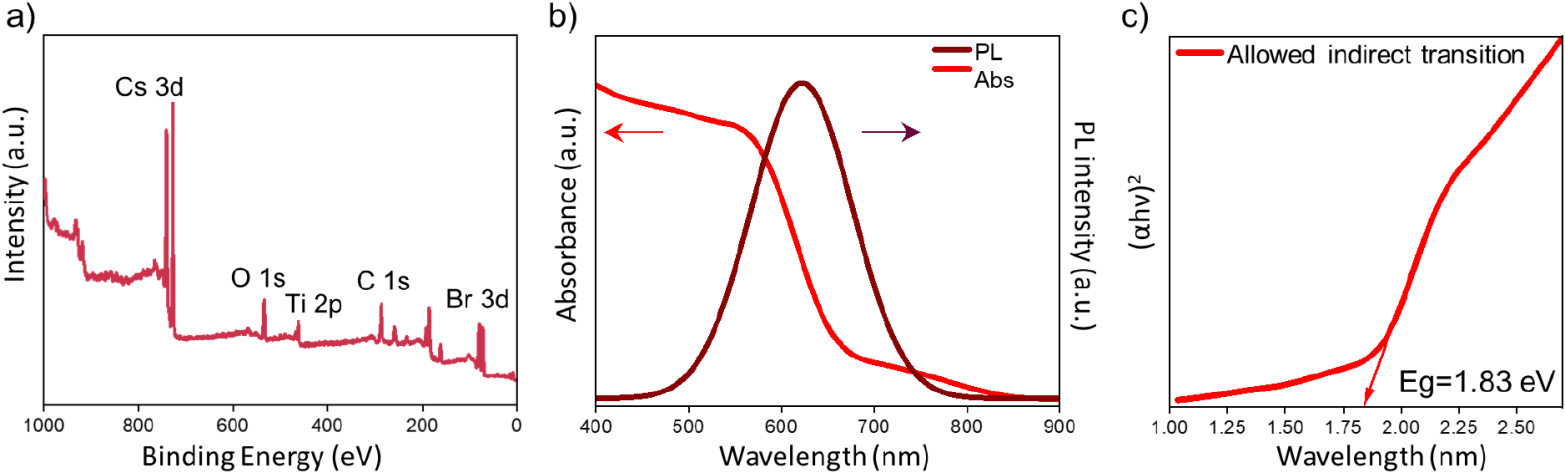
a) X-ray photoelectron spectrum (XPS) with labeled peaks, b) absorption and PL emission (λ_exc_ 375 nm) spectra, and c) Tauc plot from absorption spectrum of the MW-synthesized Cs_2_TiBr_6_ powders.

X-ray photoelectron spectroscopy (XPS) measurements of the Cs_2_TiBr_6_ powders were performed to examine the bonding characteristics and chemical states. Figure 3a presents the complete XPS spectrum, providing evidence for the presence of Cs, Ti, and Br through the sample. In Figure S6a, the Cs 3d spectrum displays two distinct peaks at energy levels of 741.8 and 727.8 eV, respectively, associated with the Cs 3d_3/2_ and Cs 3d_5/2_ orbitals. On the other hand, two distinct peaks at 468.9 and 463.1 eV can be attributed to the Ti 2p_1/2_ and Ti 2p_3/2_ orbitals, respectively (Figure S6b). Furthermore, the Br 3d spectrum (Figure S6c) exhibits two distinct peaks at 83.1 and 80.1 eV, which correspond to the Br 3d_3/2_ and Br 3d_5/2_ orbitals, respectively. These signals indicate the presence of Cs^+^, Ti^4+^ ions, and Br^–^ ions in the powder. More interestingly, the O 1s spectrum (Figure S6d) of the powder reveals three distinct peaks positioned at 536.4, 534.6, and 532.5 eV. The peak located at 536.4 eV is attributed to the presence of water molecules that have been adsorbed onto the surface of the powder material. On the other hand, the observed peaks at 534.6 and 532.5 eV are attributed to Ti–OH and Ti–O bonds, respectively. These bonds are indicative of the existence of titanium-hydroxide and titanium-oxide bonds, indicating the surface interaction of the Cs_2_TiBr_6_ powder with humidity from air (note that measurements were conducted in air).

The photoluminescence (PL) emission and absorption spectra of the Cs_2_TiBr_6_ material were performed. The emission spectrum of the Cs_2_TiBr_6_ powder after exciting at 375 nm (Figure 3b) displays a band located at 622 nm (red region) with a full width at half-maximum (fwhm) of 119.5 nm. The broadness of the emission peak is similar to values previously reported, with a 0.43 eV Stokes shift, and can be justified on the bases of the (multi-) phonon-assisted indirect transitions.^24^ The absorption spectrum (Figure 3b) shows the characteristic strong absorption of this semiconductor below 650 nm. From the Tauc Plot diagram (Figure 3c), we estimated a value for the indirect band gap of 1.83 eV.

**Figure 4:**
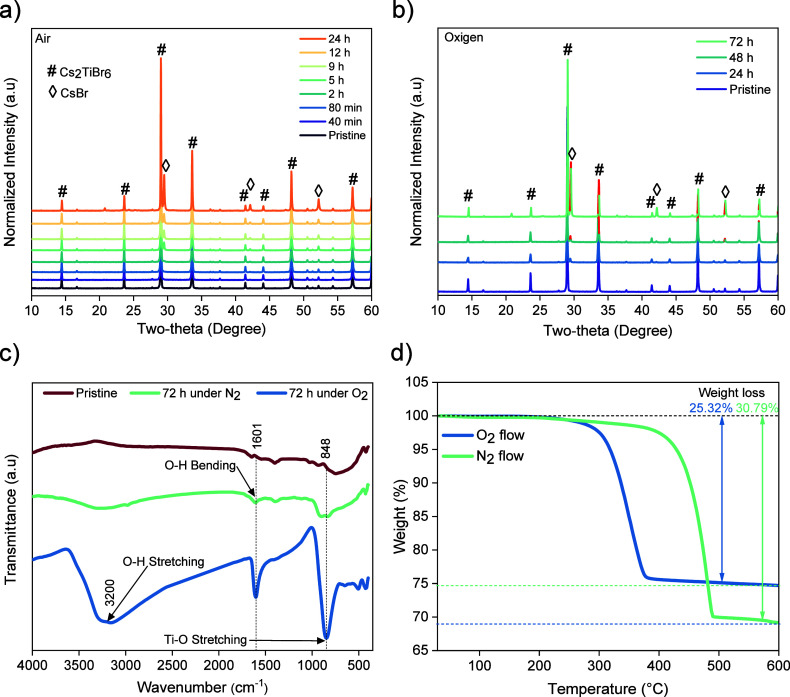
a) Evolution of XRD patterns of the Cs_2_TiBr_6_ powders over time in an ambient atmosphere (20–23 °C, 30–35% RH), b) evolution of XRD patterns of the Cs_2_TiBr_6_ powder over time in high-purity oxygen conditions, c) FTIR spectra of the pristine Cs_2_TiBr_6_ powder and after exposure to nitrogen and oxygen atmosphere, and d) thermogravimetric analysis of the Cs_2_TiBr_6_ powder under nitrogen and oxygen flows.

In order to verify the material stability, different tests have been carried out. Figure 4a shows the evolution of the XRD patterns of the Cs_2_TiBr_6_ powder in ambient air (30–35% RH, at *T* = 20–23 °C). The XRD patterns indicate that the Cs_2_TiBr_6_ powder remains unchanged after exposure to air for 40, 80, or 120 min. Only after 5 h of air exposure, the peaks associated with CsBr, as a product of the perovskite decomposition, begin to appear. This process dramatically increases after 24 h of air exposure. Unlike the results reported by other authors using a different solution synthesis,^24^ where material degradation occurs within 10 min, our optimized MW-synthesized material provided a significant improvement (30 times higher) of the stability under ambient air conditions. Indeed, the stability is comparable to the material synthesized by the group of Konstantatos et al.,^35^ in which a postsynthetic treatment with SnBr_4_ is employed to prolong the stability of Cs_2_TiBr_6_.

To verify the decomposition mechanism of the Cs_2_TiBr_6_ powder, we performed individual stability experiments under oxygen, temperature, and light conditions, as explained in subsequent subsections.

The stability of the Cs_2_TiBr_6_ powder to oxygen was investigated through its exposure to a highly pure oxygen (O_2_) environment. The XRD patterns (Figure 4b) reveal that CsBr peaks start to appear only after 24 h to O_2_ exposure. The intensity of the CsBr peaks significantly increases after 46 h of exposure, indicating that the Cs_2_TiBr_6_ powder dramatically decomposes. This phenomenon of degradation is visually observed through a gradual transformation of the powder color, transitioning from a vibrant crimson red to black as the decomposition advances. Recent mathematical models have predicted the potential decomposition of Cs_2_TiBr_6_ material over O_2._^36^

Moreover, He et al.^24^ were the first to demonstrate the instability of this material in O_2_ experimentally. Their work is consistent with our results. This may be possible given the nature of the oxidation process. Because O_2_ is a powerful oxidizing agent (: 1.23 V), Cs_2_TiBr_6_ powders can be decomposed by oxidation with O_2_ and releasing Br_2_ species (: 1.087 V). Nevertheless, in the presence of water molecules (from atmospheric moisture), the degradation of the perovskite occurs much faster. The explanation is that the highly electrophilic Ti(IV) ions react with the nucleophilic oxygen from water to produce amorphous oxo-hydroxo species of titanium. To identify the formation of these compounds, we analyzed the Cs_2_TiBr_6_ powder by FTIR spectroscopy before and after a long exposure of the material to different atmospheres (oxygen and nitrogen) (Figure 4c). The spectrum of the pristine Cs_2_TiBr_6_ powder only presents a wide weak band around 600 cm^–1^ originated from stretching Ti–Br bonds.^37^ After exposure to N_2_ for 72 h, the characteristic signals of the O–H bonds merged, presumably due to the absorption of some ambient moisture during the measurement. The two bands observed at 3200 and 1601 cm^–1^ correspond to the stretching and bending modes of O–H bonds, suggesting the presence of water or chemically adsorbed OH groups on the Cs_2_TiBr_6_ powder’s surface. In addition, a new peak centered at 848 cm^–1^ is visible. This signal is attributed to the stretching vibrations of Ti–O–Ti groups,^38^ revealing the formation of these hydroxylated titanium oxide species. After long exposure to oxygen, the bands of the hydroxyl groups and Ti–O–Ti species dramatically increase, demonstrating that the Cs_2_TiBr_6_ decomposition is facilitated under oxygen atmosphere. Theoretically, in the absence of water molecules, the decomposition of the perovskite in oxygen leads to CsBr, TiO_2_, and Br_2_ (as illustrated in eq 1). However, the moisture activates a simultaneous acid–base reaction, and amorphous Ti–O–Ti species (even hydroxylated Ti–OH-Ti bridges) are generated, as shown in FTIR measurements.
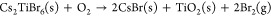
1

Thermogravimetric analysis (TGA) measurements were carried out to assess the thermal stability of the MW-synthesized Cs_2_TiBr_6_ powder. The analyses were conducted under nitrogen and air fluxes to examine the intrinsic stability and provide additional evidence for the Cs_2_TiBr_6_ reaction with oxygen to form TiO_2_ species. The thermograms (Figure 4d) show a mass loss of 2.8% under nitrogen flow and 3.7% under oxygen flow from 30 to 210 °C. These losses correspond to the evaporation of adsorbed solvent (i.e., toluene) from the material surface.^19^ The thermal decomposition of the Cs_2_TiBr_6_ powder occurs predominantly above 400 °C under N_2_ flow, which is higher than that of the conventional Pb-based perovskite (around 300 °C). In contrast, under an O_2_ flow, the system initiates its decomposition at lower temperature (300 °C), and results show that the system reacts with oxygen to produce CsBr and TiO_2_ solid. According to our results, eq 2 explains the intrinsic thermal decomposition, and the weight of the powder decreases by 8.6% under nitrogen flow and by 25.3% under oxygen flow from 100 to 400 °C. Under nitrogen flow, the powder endures a modest thermal decomposition, whereas the powder reacts with oxygen and quickly decomposes into CsBr and TiO_2_ solid. On the other hand, the weight of Cs_2_TiBr_6_ material decreases by 30.7% under nitrogen flow and 2.2% under oxygen flow at high temperatures (400–600 °C). It can be interpreted as the Cs_2_TiBr_6_ powder thermally decomposes under nitrogen flow into the CsBr solid and TiO_2_ solid, whereas the powder under oxygen flow has practically undergone oxidative decomposition in the preceding stage. The residual weight of the Cs_2_TiBr_6_ powder after the entire TGA process is 69.21%. In contrast, the remaining weight of the powder under the step of oxygen is 74.68% after complete oxidation (%), indicating that the powder inherently disintegrated into solid CsBr and gaseous TiBr_4_.

According to our results from experiments, the reaction in eq 2 can describe the intrinsic thermal decomposition of Cs_2_TiBr_6_:
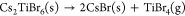
2

**Figure 5:**
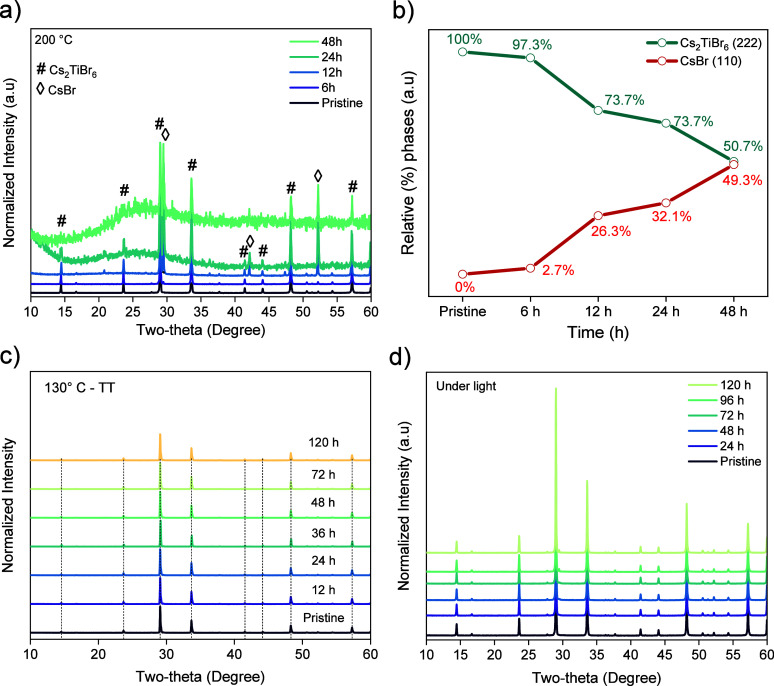
a) XRD patterns showing the effect of time over Cs_2_TiBr_6_ powders at 200 °C. b) Relative percentages of Cs_2_TiBr_6_ and CsBr phases at 200 °C after different times. c) XRD patterns showing the effect of time over Cs_2_TiBr_6_ powders at 130 °C, and d) XRD patterns of Cs_2_TiBr_6_ over time under illumination conditions.

Complementary to the TGA results, we conducted XRD measurements to provide insights into the static thermal stability. Figure 5a demonstrates that after 12 h of annealing at 200 °C in air a progressive segregation of CsBr occurs. These data are consistent with recent reports stating that Cs_2_TiBr_6_ does not decompose after 6 h of heating at 200 °C.^13,19^ The evolution of the system with time is better described in Figure 5b, which shows the relative percentage of the generated CsBr phase to the detriment of the Cs_2_TiBr_6_ perovskite. Therefore, it is demonstrated that the MW-synthesized Cs_2_TiBr_6_ powder exhibits similar instability than other reported Cs_2_TiBr_6_ materials when heated at 200 °C for long periods of time.

We also analyzed the powders’ stability at a lower heating temperature, i.e., at 130 °C, which is the temperature of the initial thermal treatment after synthesis. Figure 5c presents the XRD patterns registered after 12, 24, 36, 48, 72, and 120 h at 130 °C. Diffractograms remained basically unchanged throughout this duration, suggesting that the Cs_2_TiBr_6_ powder is stable and does not undergo significant degradation even as the heating time extended to 120 h (5 days). The Cs_2_TiBr_6_ powder’s instability at 200 °C but stability at 130 °C can be explained by the Gibbs free energy released during its decomposition. Gibbs free energy equals the enthalpy change minus the entropy change multiplied by the temperature.^39^ In this case, when the material is converted to a mixture containing gas, the entropy of the reaction products is greater than the entropy of the reactant, indicating that this is an entropy-increasing reaction.

After this study of stability, we can conclude that MW-synthesized Cs_2_TiBr_6_ powder exhibits notably enhanced thermal stability in comparison to conventional lead-based perovskites (some of them containing organic ions,^40,41^ which experience decomposition after 24 h of exposure to 85 °C in an argon atmosphere.^42,43^

An additional assessment of the powder’s stability under AM 1.5 G one sun light irradiation for periods of 24, 48, 72, 96, and 120 h was performed. The XRD patterns of all of these samples (Figure 5d) closely matched that of the pristine Cs_2_TiBr_6_ powder. This outcome underscores the remarkable light stability of the MW-synthesized Cs_2_TiBr_6_, aligning with recent findings.^14,24^ The outstanding thermal and light stability of our Cs_2_TiBr_6_ powders can be ascribed to its remarkable robust structural integrity and lower amount of structural defects.

Thus, the material generated through this synthetic approach exhibits remarkable structural stability across diverse atmospheric conditions, including exposure to air, oxygen, elevated temperatures (exceeding 130 °C), and white light. These qualities position our findings as particularly promising for applications in optoelectronics. In particular, this Pb-free perovskite material can be envisaged for novel optoelectronic devices used in eco-friendly and biofriendly environments, including solar cells and other applications (electrochromism, photochemical sensing, or photocatalysis).

## Conclusions

The synthesis of the Cs_2_TiBr_6_ double perovskite has been successfully accomplished using a rapid microwave-heating solution process. This innovative method utilizes CsBr and TiBr_4_ reagents in acetonitrile, operating under mild reaction time and temperature conditions, thereby eliminating the need for high-energy setups and vacuum systems. The reaction is greatly facilitated by employing a volatile and medium microwave-absorbing solvent. This solvent concentrates the heat conversion in the polar reagents, leading to enhanced crystallinity in the desired Cs_2_TiBr_6_ phase. As a result, the synthesized material demonstrates exceptional stability when exposed to atmospheric conditions in comparison to previous reports, surpassing the stability achieved by other methodologies by at least 30 times. Moreover, Cs_2_TiBr_6_ produced via microwave synthesis exhibits improved thermal and exposure to white light stability surpassing even lead-based halide perovskites. This research highlights that microwave-mediated solution processes offer a more scalable, sustainable, rapid, and straightforward approach for synthesizing lead-free perovskite materials, paving the way for the potential industrialization of lead-free perovskites.

## Supporting Information Available

Figure S1, (a) relative percentages and (b) average crystallite size (extracted from Scherrer equation) for the Cs_2_TiBr_6_ and CsBr phases analyzed at different conditions of MW-reaction temperature and time; Figure S2, XRD patterns showing the temperature effect over the MW synthesis (30 min); in the patterns, ◊ denotes CsBr peaks, and # represents Cs_2_TiBr_6_ peaks; Figure S3, (a) relative percentages and (b) average crystallite size (extracted from Scherrer equation) for the Cs_2_TiBr_6_ and CsBr phases analyzed at different reaction temperatures (time fixed at 30 min); Figure S4, comparison of XRD diffractograms for the optimized powders: experimental XRD pattern (in green) and simulated XRD pattern (in dark blue) developed *in silico* using DFT calculations and VASP code for the represented Cs_2_TiBr_6_ crystal structure; Figure S5, (a) SEM photograph of Cs_2_TiBr_6_ powder, the scale bar represents 1 μm, (b) EDS spectrum of the MW-synthesized Cs_2_TiBr_6_ powder including the Cs:Ti:Br atomic percentage, which fits well with Cs_2_TiBr_6_ element proportion; Figure S6, (a) XPS spectra of the Cs_2_TiBr_6_ powder exhibit a high level of resolution for (a) Cs 3d, (b) Ti 2p, (c) Br 3d, and (d) O 1s (including deconvolution of the different oxygen species present in the sample) (PDF)
