# Correction to “Microwave-Mediated Synthesis of Lead-Free Cesium Titanium Bromide Double Perovskite: A Sustainable Approach”

**DOI:** 10.1021/acs.chemmater.4c02554

**Published:** 2024-09-25

**Authors:** Emmanuel Reyes-Francis, Carlos Echeverría-Arrondo, Diego Esparza, Tzarara López-Luke, Tatiana Soto-Montero, Monica Morales-Masis, Silver-Hamill Turren-Cruz, Iván Mora-Seró, Beatriz Julián-López

The authors regret an error in the acknowledgments section of the original article. A correction has now been made. The previously stated sentence read: “S.H.T.C would like to thank the Spanish Ministry of Economy, Industry and Competitiveness (postdoctoral contract Juan de la Cierva Formación FJC2019-041835-I) and POLONES BIS 1 (DEC-2021/43/P/ST5/01780) for the financial support during this work.” A sentence was inadvertently omitted in the published version. The corrected acknowledgment now reads: “S.H.T.C would like to thank the Spanish Ministry of Economy, Industry and Competitiveness (postdoctoral contract Juan de la Cierva Formación FJC2019-041835-I) and POLONEZ BIS project No. 2021/43/P/ST5/01780 co-funded by the National Science Centre and the EU’s H2020 research and innovation programme under the MSCA 945339 for the financial support during this work.” This revision does not affect the main text and the conclusion of the published article. The authors would like to apologize for any inconvenience caused.

