# Supplementary material for: Microwave-Mediated Synthesis of Lead-Free Cesium Titanium Bromide Double Perovskite: A Sustainable Approach
Source: Chem Mater. 2024 Feb 2;36(3):1728–36. doi: 10.1021/acs.chemmater.3c03108 (PMC10870712; doi:10.1021/acs.chemmater.3c03108)
Supplement: Supplementary file 1 — cm3c03108_si_001.pdf [file cm3c03108_si_001.pdf]

SUPPORTING INFORMATION

Microwave-mediated Synthesis of Lead-Free Cesium Titanium Bromide Double Perovskite: A Sustainable Approach

Emmanuel Reyes-Francis,[1,2] Carlos Echeverría-Arrondo,[2] Diego Esparza,[3] Tzarara López-Luke,[1] Tatiana Soto-Montero,[4] Monica Morales-Masis,[4] Silver-Hamill Turren-Cruz,[2,5,] Iván Mora-Seró,[2,*] and Beatriz Julián-López[2,*]*

[1] Instituto de Investigación en Metalurgia y Materiales, Universidad Michoacana de San Nicolás de Hidalgo, Edificio U, Ciudad Universitaria, Morelia, Mich, C.P. 58030, Mexico.

[2] Institute of Advanced Materials (INAM), Universitat Jaume I, Av. Sos Baynat, s/n, 12071, Castelló de la Plana, Spain.

[3] Unidad Académica de Ingeniería Eléctrica, Universidad Autónoma de Zacatecas. Jardín Juárez 147, Zacatecas Centro, C.P. 98000, Zacatecas, Mexico.

[4] MESA+ Institute for Nanotechnology, University of Twente, Enschede, 7500 AE, The Netherlands

[5] Department of Physical Chemistry, Polish Academy of Sciences, Warsaw 01-224, Poland

Supporting Information file contents:

Figure S1. a) Relative percentages and b) average crystallite size (extracted from Scherrer equation) for the Cs_2TiBr_6 and CsBr phases analyzed at different conditions of MW-reaction temperature and time.

Figure S2. XRD patterns showing the temperature effect over the MW synthesis (30 min). In the patterns, ◊ denotes CsBr peaks, and # represents Cs_2TiBr_6 peaks.

Figure S3. a) Relative percentages and b) average crystallite size (extracted from Scherrer equation) for the Cs_2TiBr_6 and CsBr phases analyzed at different reaction temperatures (time fixed at 30 min.

Figure S4. Comparison of XRD diffractograms for the optimized powders: experimental XRD pattern (in green) and simulated XRD pattern (in dark blue) developed *in silico* using DFT calculations and VASP code for the represented Cs_2TiBr_6 crystal structure.

Figure S5. a) SEM photograph of Cs_2TiBr_6 powder, the scale bar represents 1 μm, b) EDS spectrum of the MW-synthesized Cs_2TiBr_6 powder including the Cs:Ti:Br atomic percentage, which fits well with Cs_2TiBr_6 element proportion.

Figure S6. a) XPS spectra of the Cs_2TiBr_6 powder exhibit a high level of resolution for a) Cs *3d*, b) Ti *2p*, c) Br *3d*, and d) O *1s* (including deconvolution of the different oxygen species present in the sample).

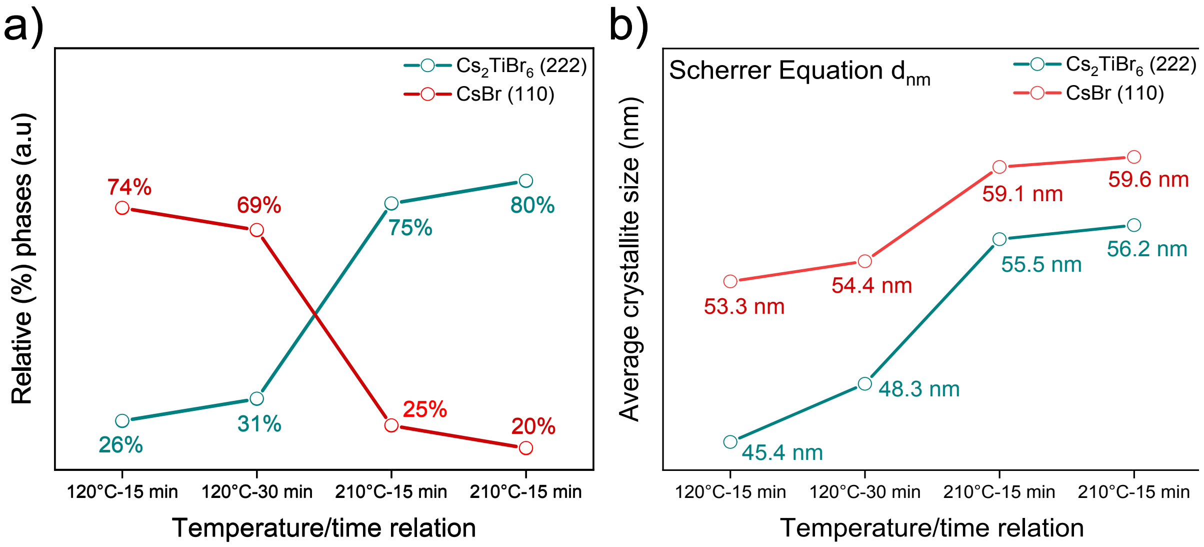

Figure S1. a) Relative mass percentages calculated from XRD patterns (*) and b) average crystallite size (extracted from Scherrer equation) for the Cs_2TiBr_6 and CsBr phases analyzed at different conditions of MW-reaction temperature and time.

(*) X-ray diffraction technique allows quantitative analysis of a crystalline phase (component P) in a solid sample by adding an internal standard s (internal standard method [1]). The formula developed by Alexander and Klug is:

$$x_p = kI_p/I_s$$

where k is a coefficient related to the nature of component P and internal standard s, to properties of the X-ray diffraction measurement (i.e., the geometry of the apparatus and the wavelength of the X-ray) and to the amount added from the standard. x_p is the mass % of component P. I_p and I_s are the X-ray diffraction intensities of the more intense hkl peaks of component P and the standard s, respectively.

For the quantitative analysis of our binary mixtures composed of Cs_2TiBr_6 and CsBr, we prepared a set of samples using corundum as standard and CsBr as component P. The experimental XRD patterns provided us with the intensities of the peaks of corundum and CsBr (we considered (104) and (110) planes, respectively) and k was extracted from the equation. Then, we added 10% mass of corundum to our binary mixtures and, with the experimental peak intensities and k, we calculated the mass % of CsBr (x_{CsBr}). The mass % of Cs_2TiBr_6 was directly inferred because $x_{Cs2TiBr6} + x_{CsBr} = 1$ (or 100 % in percentage). The representation of the mass % of both phases is given in Figures S1 and S3.

[1] L. Alexander, H.P. Klug, Basic aspects of X-ray absorption in quantitative diffraction analysis of powder mixtures, Anal. Chem., 20 (1948), 886-889.

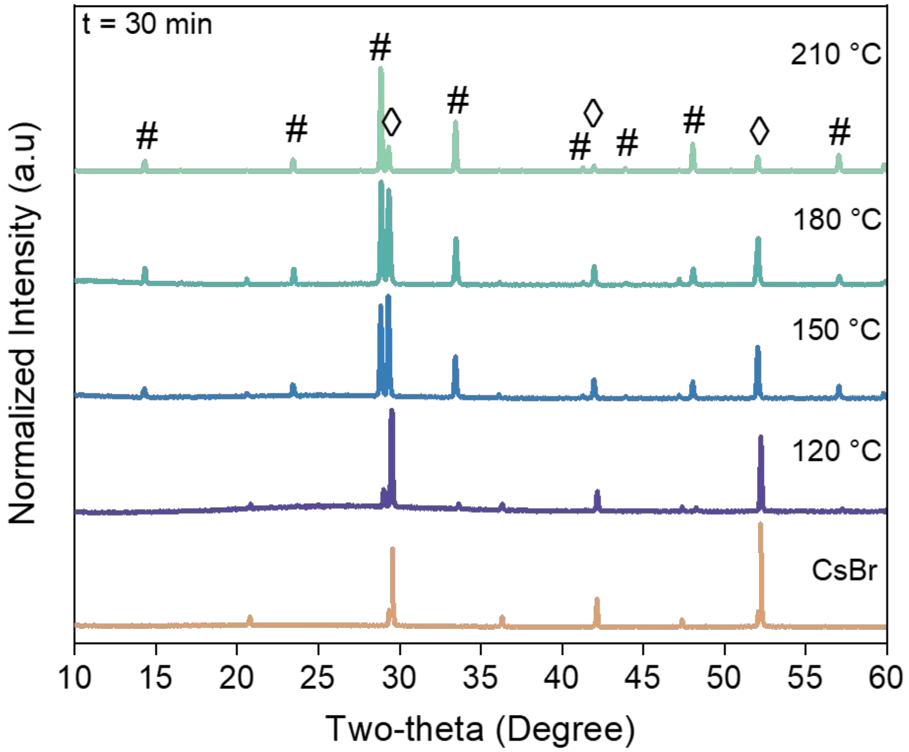

Figure S2. XRD patterns showing the temperature effect over the MW synthesis (30 min). In the patterns, ◊ denotes CsBr peaks, and # represents Cs_2TiBr_6 peaks.

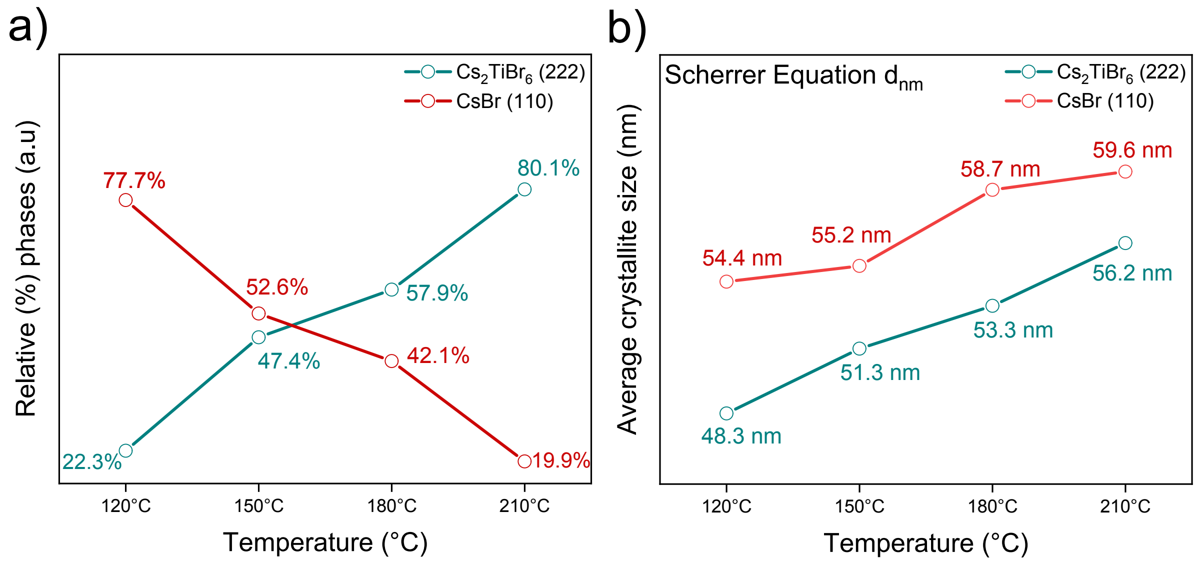

Figure S3. a) Relative mass percentages calculated from XRD patterns and b) average crystallite size (extracted from Scherrer equation) for the Cs_2TiBr_6 and CsBr phases analyzed at different reaction temperatures (time fixed at 30 min.

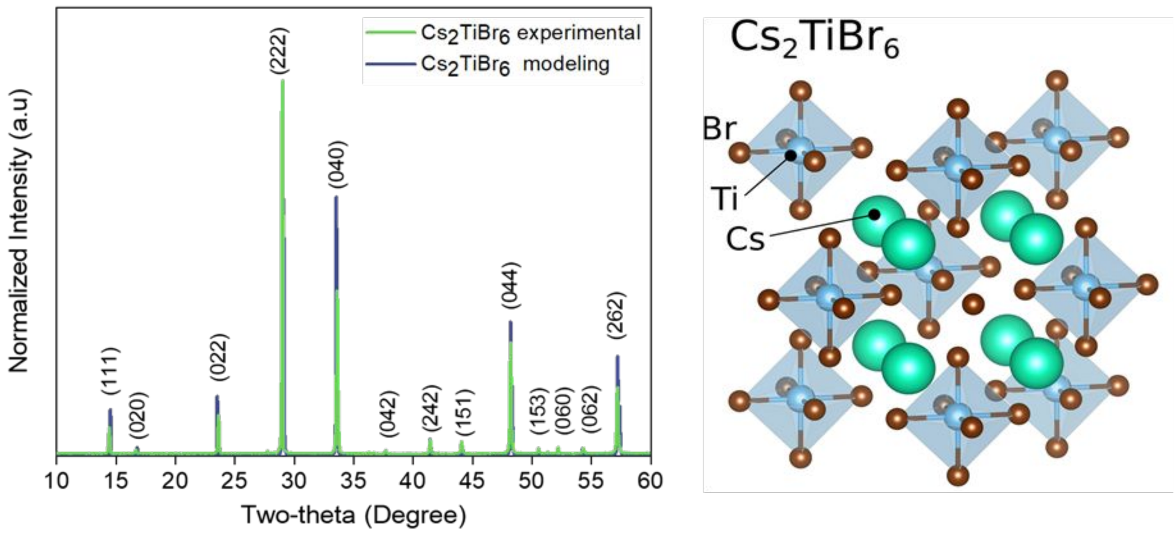

Figure S4. Comparison of XRD diffractograms for the optimized powders: experimental XRD pattern (in green) and simulated XRD pattern (in dark blue) developed *in silico* using DFT calculations and VASP code for the represented Cs_2TiBr_6 crystal structure.

a) b)

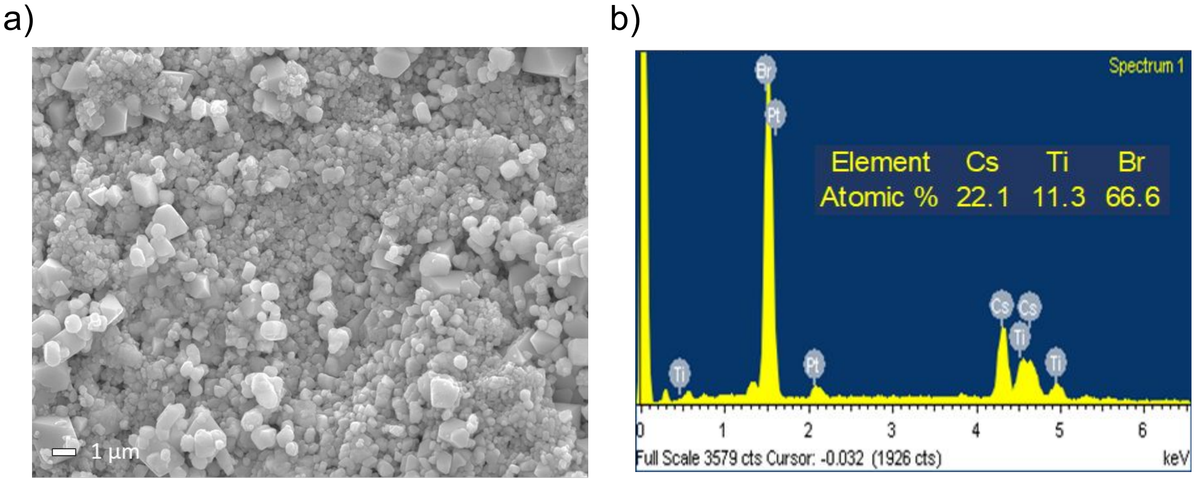

Figure S5. a) SEM photograph of Cs_2TiBr_6 powder, the scale bar represents 1 μm, b) EDS spectrum of the MW-synthesized Cs_2TiBr_6 powder including the Cs:Ti:Br atomic percentage, which fits well with Cs_2TiBr_6 element proportion.

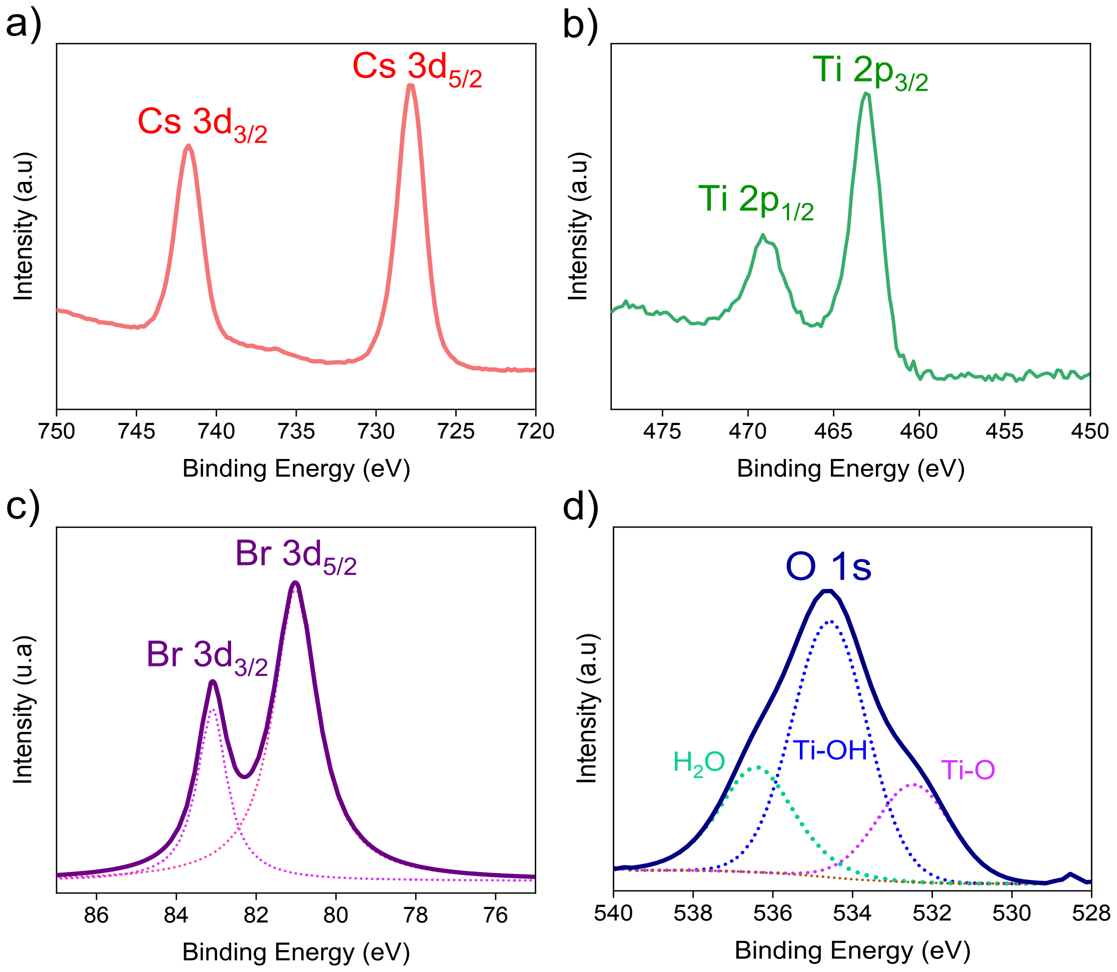

Figure S6. a) XPS spectra of the Cs_2TiBr_6 powder exhibit a high level of resolution for a) Cs *3d*, b) Ti *2p*, c) Br *3d*, and d) O *1s* (including deconvolution of the different oxygen species present in the sample).
